# Liver Transplantation for Hepatocellular Carcinoma beyond Milan Criteria: Multidisciplinary Approach to Improve Outcome

**DOI:** 10.1155/2014/706945

**Published:** 2014-03-04

**Authors:** A. Kornberg

**Affiliations:** Department of Surgery, Klinikum rechts der Isar, Technical University Munich, Ismaningerstraße 22, D-81675 Munich, Germany

## Abstract

The implementation of the Milan criteria (MC) in 1996 has dramatically improved prognosis after liver transplantation (LT) in patients with hepatocellular carcinoma (HCC). Liver transplantation has, thereby, become the standard therapy for patients with “early-stage” HCC on liver cirrhosis. The MC were consequently adopted by United Network of Organ Sharing (UNOS) and Eurotransplant for prioritization of patients with HCC. Recent advancements in the knowledge about tumor biology, radiographic imaging techniques, locoregional interventional treatments, and immunosuppressive medications have raised a critical discussion, if the MC might be too restrictive and unjustified keeping away many patients from potentially curative LT. Numerous transplant groups have, therefore, increasingly focussed on a stepwise expansion of selection criteria, mainly based on tumor macromorphology, such as size and number of HCC nodules. Against the background of a dramatic shortage of donor organs, however, simple expansion of tumor macromorphology may not be appropriate to create a safe extended criteria system. In contrast, rather the implementation of reliable prognostic parameters of tumor biology into selection process prior to LT is mandatory. Furthermore, a multidisciplinary approach of pre-, peri-, and posttransplant modulating of the tumor and/or the patient has to be established for improving prognosis in this special subset of patients.

## 1. Introduction

Hepatocellular carcinoma (HCC) is the most frequent primary malignant tumor of liver cells [1–3]. Disease burden owing to HCC is significantly increasing in recent years. It is currently the fifth most common cancer and the third most common reason for cancer-related mortality worldwide [1–6]. HCC mainly occurs in a damaged organ; liver cirrhosis as a result of viral hepatitis (hepatitis B virus (HBV) or/and hepatitis C virus infection (HCV)) or chronic alcohol abuse is a major risk factor for development of HCC. The incidence of viral hepatitis is markedly increasing worldwide, which will even enhance the epidemiologic importance of HCC in the near future [7–10].

Continuous clinical surveillance programs in patients with liver cirrhosis were shown to be useful in the detection of HCC at early stages. Recommended surveillance strategies are based on periodic evaluation by ultrasound imaging and determination of blood levels of the tumor marker alpha-fetoprotein (AFP) [11–14]. Suspicious intrahepatic lesions should be further evaluated by advanced imaging techniques such as contrast-enhanced ultrasound and computed tomography (CT) and/or magnetic resonance tomography (MRI). Based on their imaging characteristics, such as arterial hypervascularity and early wash-out phenomenon in the portal phase, lesions of more than 2 cm can be well detected and described [15–18]. Histopathologic differentiation may be necessary in lesions smaller than 1-2 cm. Although percutaneous tumor biopsy carries a small risk of bleeding and tumor seeding, it provides useful information about biological tumor aggressiveness, such as grading, microvascular tumor invasion (MVI), and molecular markers [19–21].

Liver resection (LR), liver transplantation (LT), and percutaneous tumor ablation are currently considered as curative treatment options for HCC in different stages of disease. Hepatic resection is the traditional treatment of choice in patients with HCC in noncirrhotic livers, which accounts for about 5% of cases in the western and about 40% of cases in the eastern world, respectively [22–25]. Major LR by conventional or extended hemihepatectomy may currently be performed with relatively low rates of serious complications. In this clinical constellation, early postoperative mortality is mainly determined by functional liver reserve after resection. Hence, all types of tumors may be surgically removed, as long as sufficient functional liver reserve will remain and support a beneficial clinical course. Some large series have recently demonstrated 5-year survival rates between 30% and 50% in this clinical setting [22–27].

In contrast, resection of HCC in cirrhotic patients is still a high-risk surgical procedure requiring an interdisciplinary expert selection process of suitable candidates. In this context, exact functional evaluation of cirrhotic damage and portal hypertension is mandatory, since both are well-known major determinants for postoperative morbidity and mortality [23, 28–30]. In recent years, significant proceedings in pre-, intra-, and postoperative management of cirrhotic patients have remarkably improved prognosis. Adequate patients' selection, exact preoperative radiographic planning, and tumor reduction by interventional neoadjuvant procedures have been identified as useful neoadjuvant strategies [31–33]. Apart from that, the functional remnant liver volume after LR may be significantly increased by preoperative portal vein embolization. This procedure should be discussed, when estimated functional remnant liver volume is less than 40% of the calculated total liver volume [33–36]. The combination of intraoperative ultrasound, gentle dissection techniques, and anatomic resection approaches and the application of intermittent inflow occlusion have significantly reduced intraoperative trauma of the liver tissue [37–40]. In addition, postoperative intensive care management was optimized in recent years [41, 42].

As a result of these clinical advancements, perioperative mortality after LR in cirrhotic patients has decreased from about 15% in the 1980s to about 5% nowadays [43–48]. Some centers even reported about zero mortality in highly selected patients, such as the Barcelona group in 100 patients with single HCC nodules but without suffering portal hypertension [49].

However, based on the two most common liver function algorithms, resectability rate is still very low (5% to 10%). Makuuchi et al. presented in 1986 and 1995 an algorithm of indication based on three clinical variables: ascites, bilirubin, and indocyanine green test [50, 51]. It is widely accepted that nontreatable ascites, elevated bilirubin level, and increased ICG retention exclude patients from extended liver resections. The Barcelona Clinic Liver Cancer (BCLC) group identified the absence of clinically relevant portal hypertension and normal bilirubin level as major determinants for beneficial outcome after LR [49, 52]. It is easy to imagine that, based on these clinical algorithms, a majority of cirrhotic patients with HCC are no suitable candidates for LR.

A high rate of tumor recurrence remains another key issue in the context of LR for HCC. Between 10%–50% and 70% of patients will develop tumor recurrence at 2 and 5 years, either as intrahepatic metastases from primary HCC (true recurrence) or as development of de novo tumors on remaining cirrhotic liver tissue [46–49, 53]. Multiple tumor nodules, vascular tumor invasion, and the presence of tumor satellites were identified as major predictive factors for tumor relapse after LR. The identification of these risk factors at histopathologic evaluation should, therefore, intensify postoperative surveillance programs [54–56].

Percutaneous radiofrequency ablation (RFA) and LT are options of curative care for those patients with HCC that are not eligible for liver resection due to associated diseases [57, 58]. Although RFA is recommended in tumors less than 5 cm, the probability of complete tumor necrosis is the highest for smaller tumors (<2 cm). Apart from tumor size, efficacy of RFA is limited by tumor site. In the case of tumors overhanging the liver margin and approaching adjacent organs, such as the gallbladder, stomach, or colon, or when tumor is located nearby major intrahepatic vessels, RFA may be technically unfeasible due to risk of thermal injuries [59–63].

### 1.1. Rationale for Liver Transplantation in Patients with HCC

In contrast to LR and RFA, LT has the potential to eliminate HCC and the underlying tumor-generating cirrhosis. It provides the widest possible surgical margin and is, therefore, able to reduce the risk of tumor recurrence. In addition, it restores normal liver function. From an oncological and functional point of view, LT may be the optimal treatment for HCC in cirrhotic patients. However, life-time need of immunosuppressive therapy with persistent risk of tumor recurrence and significant shortage of adequate donor organs are relevant limitations of this therapeutic option [64–66].

LT for HCC is as old as LT itself. The early series of Thomas E. Starzl from Pittsburgh consisted of 4 patients, including 2 children in the 60s with the longest survival of 16 months [67]. Basically, the role of LT in the treatment of HCC has evolved over consecutive periods. During the 80s, high tumor recurrence rates (32%–54%) and low survival rates (5-year survival 20%–40%) were reported to result from accepting advanced tumor stages with regard to macromorphology (number and size of tumor nodules) and biology (poor tumor differentiation, macrovascular invasion, lymph node involvement, and extrahepatic spread) [68–72]. At the same time, waiting time for a suitable donor organ increased to more than 1 year in many transplant centers making LT inappropriate for treatment of HCC, since this was by far exceeding median survival prognosis of the patients. In view of extraordinary treatment costs and lack of organ availability, these poor outcome data have globally questioned the justification of LT in patients with HCC. It prompted the US Department of Health and Human services to declare HCC as contraindication for LT [73].

The second period of development started at the early 90s, when data reassessment suggested that patients with incidental and asymptomatic HCC may achieve outcome results that are comparable to patients with nonmalignant liver disease. Bismuth and colleagues reported in 1993 about survival benefit after LT versus LR in patients with small uninodular or binodular tumors (<3 cm). Apart from that, the authors identified diffuse tumor manifestation, more than two tumor nodules >3 cm, and presence of portal tumor thrombus as high risk factors for tumor recurrence [74]. In a landmark manuscript, Mazzaferro and colleagues reported in 1996 about excellent outcome in 48 liver transplant patients having a single HCC nodule of 5 cm or less or a maximum of 3 tumor nodules, each with a maximum diameter of 3 cm [75]. Twenty-eight of them underwent preoperative therapy, mainly by transarterial chemoembolization (TACE). Patients with evidence of macrovascular invasion or lymph node involvement were excluded. In detail, patients with HCC meeting these so-called Milan criteria (MC) were able to achieve a 4-year survival rate of extraordinary 75%, which was not different from LT for nonmalignant disease. Patients who fulfilled these criteria on basis of explant histopathology achieved an actuarial survival of 85% after 4 years. It was, however, equally noteworthy that 50% of patients were alive after 4 years, although tumors were exceeding the MC. The authors have critically discussed the issue of understaging histopathologic tumor stage by radiographic imaging [75]. Many studies have subsequently confirmed the paramount prognostic value of the MC in the transplant setting [64, 76–82]. In consequence, LT was since considered as first line therapy for early HCC in liver cirrhosis around the world. Furthermore, vascular tumor infiltration and poor tumor grading were assessed as strong predictors of tumor recurrence and poor outcome [82–84]. The successful implementation of the MC in the nineties is not only consequence of limiting tumor size for indication but also a result of selecting favourable tumor stages by short-term waiting times of less than 6 months. The risk of prognostic relevant tumor progression remained low at that time. Furthermore, the transplant community has defined an expected survival probability of 50% for qualifying patients to be listed for LT, which is still consensus until these days [4]. In the latest period of development beginning in the 2000s and still lasting, we are currently facing another challenge, characterized by discrepancy between increasing numbers of liver transplant candidates on the one hand and by significant shortage of appropriate donor organs on the other hand [85]. Waiting times prior LT have significantly prolonged and drop-out rates have remarkably increased in recent years, finally resulting in deterioration of survival rates as based on an intent-to-treat basis [76–78]. Increasing waiting times implicate that many patients meeting the MC at listing will exceed them prior to LT. However, with exception of macrovascular tumor invasion and extrahepatic tumor spread, there are currently no consistently accepted criteria for tumor-related patient delisting/drop-out [76–79].

With other words, LT has become a victim of its own success. The MC are confronted by a critical reappraisal with special regard to excluding patients with special regard to exclude patients from curative treatment due to advanced HCC stages, although tumor biology might be favourable [85–87]. In recent years, a huge number of studies have suggested extended criteria that may offer a minimum cut-off survival probability of 50% after 5 years [87–100]. Some of them are listed in Table 1.

### 1.2. The Milan Criteria and “Beyond”

The MC have incorporated features of tumor macromorphology, such as size and number of HCC nodules, into the same definition of “early HCC” [75, 82]. Patients with HCC meeting them (one solitary tumor nodule up to a maximum of 5 cm or a maximum of 3 HCC nodules and each of them up to a maximum diameter of 3 cm, without macrovascular invasion and extrahepatic tumor spread) were demonstrated to achieve excellent long-term survival rates [75–79]. Therefore, these criteria became worldwide “standard” for patient selection process prior to LT. The United Network for Organ Sharing (UNOS) database analysis including 48887 liver transplant patients with HCC between 1987 and 2001 has shown that implementation of the MC led to a significant improvement of survival from 25.3% (1987–1991) to 61.1% (1996–2001) within two decades [101]. The definition of “early HCC” as based on the MC has modulated innovative classification systems of HCC such as the new TNM system and the BCLC (Barcelona Clinic Liver Cancer) classification [19, 102].

As logical consequence, MC were incorporated into the modified liver allocation systems of UNOS in 2001 and Eurotransplant in 2006. Both regions are currently using the model for end-stage liver disease (MELD) score-based prioritization system. It is mainly based on three laboratory values (international normalized ratio, bilirubin, and creatinine) and provides exceptional priority upgrades for several indications, such as HCC meeting the MC [103–105]. The MELD score was demonstrated to predict waiting list mortality in patients with end-stage liver disease. A higher MELD score implies an increased risk of mortality on the waiting list during the next three months. However, liver transplant candidates with HCC have often a rather low MELD score, as they frequently suffer from Child A cirrhosis. Therefore, these patients experience a continuous MELD score upgrade in order to increase their chance for receiving a liver allograft in time [105]. Only patients with HCC meeting the MC qualify for repeated MELD score upgrading [106–110]. Macromorphologic tumor progression beyond the Milan size limits results in loss of MELD prioritization. This, however, does not automatically imply drop-out from the waiting list. Tumor-related patients' removal is, in the last analysis, a decision of the transplant center, not at least based on biological tumor behaviour. The transplant team might come to the conclusion that the patient is still suitable for LT by rescue allocation or living donor liver transplantation (LDLT) [108–110]. Currently, the only uncontroversially accepted tumor-related drop-out criteria are macrovascular tumor invasion and extrahepatic tumor spread [4, 7, 64, 65].

With increasing experiences in recent years, limitations of radiographic imaging techniques to exactly predict histopathologic tumor staging became obvious. A high number of explanted livers have demonstrated HCC beyond MC, although final clinical evaluation staged them within MC [65, 66, 76]. Nodules in cirrhosis may have regenerative, dysplastic, or malignant character or overlapping. For nodules of less than 2 cm, radiographic imaging often shows no hypervascularization, the hallmark of HCC [15–18, 111]. Percutaneous biopsy is often necessary but not always possible. Under- and overestimation rates between 20% and 40% have been reported [76–79]. Nonetheless, many of the patients with HCC beyond MC on explant histopathology were alive after 5 years, which has retrospectively justified the procedure. In many trials, 5-year recurrence-free survival rates above 50% were reported in patients with HCC beyond MC on explant histopathology [80–82, 112]. This outcome phenomenon has additionally heated the critical review of the MC. Against this background, huge efforts are being made to improve imaging techniques and to identify features of biological tumor behaviour that may be usefully implemented in decision process [78–100]. It is evident that a multidisciplinary approach of modulating tumor biology and the patients' vulnerability for HCC relapse in the pre-, peri-, and posttransplant period is mandatory for achieving acceptable outcome in this special subset of patients. The aim of this review is to report on multidisciplinary options that are currently available to establish LT as curative treatment for HCC beyond MC.

## 2. Pretransplant Approach: Patient Selection and Neoadjuvant Tumor Treatment

### 2.1. Patient Selection

The aim of extending the selection criteria for LT is to provide more patients the chance of being cured from HCC. Considering the increasing shortage of adequate donor organs, the liver transplant candidates selected according to extended criteria must still have an acceptable probability of long-term survival. Although reliable data on this subject is still rare, a minimum of 50% survival likelihood at 5 years after LT is consistently demanded in this clinical setting [75–100]. A huge number of new expanded criteria systems have been proposed in recent years (Table 1). However, comparability of respective data is very limited for several reasons. First, most of the trials are of retrospective character. Second, the numbers of patients included, the immunosuppressive regimens used, the applied neoadjuvant concepts, and the drop-out criteria vary considerably. Third, few of the trials are performed on an intent-to-treat approach, while others were not. Fourth, in many series, patients underwent deceased donor LT (DDLT), while LDLT was the preferred procedure in other studies. And, furthermore, there are great inconsistencies on whether the established criteria were based on pretransplant imaging findings or on results of explant histopathology (Table 1) [75–115]. Expansion of transplant criteria based on macromorphologic tumor stage was the most consistent approach. Yao et al. from the University of California, San Francisco (UCSF), were the first to describe a modest expansion of tumor size beyond MC in their landmark manuscript from 2001 [88]. They reported about excellent 5-year survival rates in patients with single tumors up to 6 cm or up to 3 tumor nodules, each ≤4.5 cm in diameter and total tumor volume not exceeding 8.5 cm, without cross vascular invasion. For tumours exceeding the so-called UCSF criteria on explant histopathology, 1- and 5-year recurrence-free survival rates were 80.4% and 59.5% compared to 98.6% and 96.7% in those with HCC meeting them. At the first glance, the UCSF criteria seem to be safe and to guarantee excellent outcome data that are comparable to conventional MC. Adopting the UCSF criteria, an additional 5%–20% of HCC patients might be considered as suitable liver transplant candidates [88, 116, 117].

However, its implementation was accompanied by some critical comments. First, it is a retrospective data analysis. Second, patients' characteristics are very heterogeneous since several of them underwent pretransplant TACE for tumor downstaging, while others did not. Third, it is a histopathology-based analysis, which does not correspond to clinical decision process. And above all, the number of patients meeting the UCSF criteria but exceeding the Milan size limits was rather small [88, 117–119]. Thus, the UCSF data have rather been judged as appreciation of the MC than as the establishment of a novel and feasible expanded criteria system [117–119]. The overlapping subset of patients meeting the UCSF but exceeding the MC frequently accounts for <10% of the transplanted population. This has especially been pointed out by a large French retrospective multicenter trial [120]. Based on explant histopathology reviews, the authors reported on 184 Milan In patients and 238 UCSF Out patients but only 39 patients exceeding the Milan but meeting the UCSF criteria (8.7%), respectively [120]. The 5-year overall survival rates were 70.4%, 63.6%, and 34.1% in Milan In patients, Milan Out/UCSF In recipients, and UCSF Out patients, respectively (*P* < 0.001). The outcome was comparable (*P* = 0.13) between Milan In and Milan Out/UCSF In recipients. In an intent-to-treat analysis, however, as based on clinical staging, corresponding 5-year overall survival rates were only 60.1%, 45.5%, and 34.7%, respectively (*P* < 0.001). The critical issue of insufficient pretransplant tumor staging by imaging techniques is adequately addressed in this trial. This has to be taken into consideration in all pathology-based trials and is still a major concern for liberalization of conventional criteria systems [120]. The UCSF group have prospectively validated their extended criteria system by clinical staging in 138 liver transplant patients over a period of 5 years [121]. Five-year recurrence-free survival rates after LT were 90% and 93% in patients meeting the MC and exceeding them but meeting the UCSF criteria, respectively. Understaging by preoperative imaging was reported in up to 28% of the cases [121]. Recently, Duffy and colleagues from the University of California Los Angeles (UCLA) reported about the largest single institution experience with LT in 467 patients with HCC [92]. They did not find a statistical difference in 5-year survival between patients meeting MC and patients beyond Milan but meeting UCSF criteria by both, preoperative imaging (79% versus 64%; *P* = 0.061) and explant histopathology (86% versus 71%; *P* = 0.057), respectively. In contrast, 5-year survival rates were below 50% in patients with HCC exceeding the UCSF criteria. This trial is powered by a high number of patients exceeding MC but meeting the UCSF size limits (*n* = 185 by preoperative imaging; *n* = 208 by explant histopathology). Multivariate analysis identified number of tumor nodules, lymphovascular invasion, and poor differentiation as independent predictors of mortality. The authors have, therefore, concluded that selection criteria may be safely expanded to the UCSF burden limits without negatively affecting outcome [92]. Nevertheless, there was a clear trend of survival deterioration in the “extended” subpopulation. This, however, may be still accepted, since 5-year survival exceeded the critical cut-off value of 50% [92].

**Figure 1:**
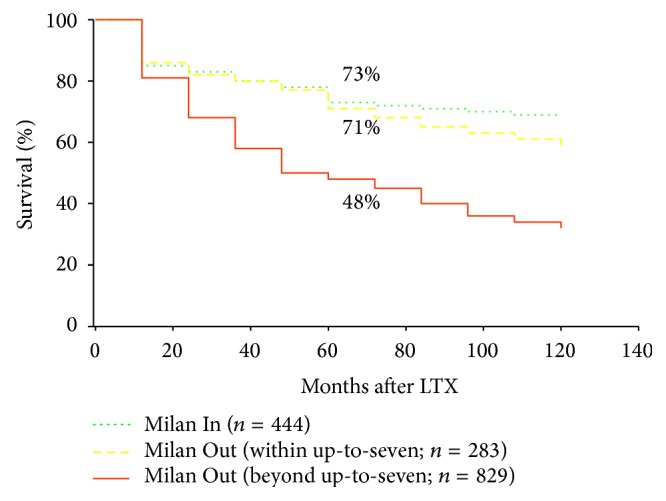
Patients meeting the up-to-seven criteria had a comparable survival rate (71.2%) than patients meeting the MC (73.3%) (adapted from [98]).

In a retrospective multicenter trial, the Milan group have recently reported about the outcome in 1112 liver transplant patients with HCC exceeding the MC at histopathology reviews compared to 454 patients with HCC meeting them (the so-called Metroticket project) [98]. Five-year overall survival for those patients exceeding them was 53.6% compared to 73.3% for those that met the criteria. The authors identified a subgroup of 283 patients without microvascular invasion but meeting the new created so-called “up-to-seven” criteria (HCC with seven as the sum of maximum size of the largest tumor in cm and the number of tumors) that achieved an excellent 5-year survival of 71.2%, which was similar to the Milan In population (Figure 1). Furthermore, they demonstrated a linear effect of hazard ratios with tumor size, whereas the effect tended to stagnate for tumor numbers above 3. Based on these data, the Milan group created the “Metroticket calculator” (HCC forecast chart), aiming at assessment of 5-year survival probability based on size and number of tumor nodules (http://www.hcc-olt-metroticket.org/calculator/). It clearly illustrates that the expansion of the selection criteria beyond the Milan burden limits may be paid by an increased risk of tumor recurrence and reduced survival. The core issue is whether and how far we can afford a limited survival probability in transplanting more advanced HCC.

These trials clearly elucidated that extended selection criteria as based on macromorphology have to be augmented by features of biological tumor behaviour, such as tumor grading and MVI, in order to limit the expected risk of HCC recurrence [70–120].

Exclusion of poorly differentiated tumors by pretransplant biopsy is discussed as one approach of safely expanding macromorphologic criteria for LT. Cillo and colleagues from the University of Padua reported about their outcome data when accepting any tumor size but excluding poorly differentiated tumors by pre-LT biopsy [94]. Finally, a total of 48 patients with HCC (15 of them incidentally diagnosed) were included, implementing TACE as neaoadjuvant locoregional therapy. On histopathologic examination, 38% of the study group were beyond MC. The authors reported about an excellent overall and recurrence-free survival after 5 years of 75% and 92%, respectively. The same group presented in 2007 data of an intent-to-treat analysis including 100 patients listed for LT based on this selection process [122]. Forty of them exceeded the MC, while 60 liver transplant candidates continued to meet them. All patients underwent a specific and aggressive multimodal neoadjuvant treatment algorithm while waiting for LT. The cumulative 6- and 12-month drop-out probabilities were 0% and 4% for Milan Out, and 6% and 11% for Milan In recipients, respectively. The 1- and 3-year survival rates on an intent-to-treat basis were not different between patients meeting (84%, 69%) and those exceeding (95% and 85%) the MC. Notably, the authors did not recognize tumor seeding or any other complication by percutaneous tumor biopsy, which is in contrast to several other trials [122–125]. And, recently, DuBay et al. from Toronto reported about comparable overall 5-year survival rates between 189 patients meeting the MC (72%) and 105 liver recipients exceeding them (70%), when imaging studies and pretransplant biopsy ruled out macrovascular invasion and poor tumor differentiation and an aggressive neoadjuvant treatment concept has been implemented [100].

Although data of these trials is rather optimistic, there remains an obvious risk of tumor seeding and false negative biopsy findings, which seems to limit wide applicability of pretransplant tumor biopsy for patient selection [122–125]. In particular, MVI, another prognostic relevant parameter of tumor aggressiveness, may not accurately be assessed by pre-LT biopsy but only at explant histopathology [126–128]. Furthermore, vascular invasion may be part of significant tumor progression while waiting for LT, which will not be adequately reflected in biopsy findings at patients' listing [126–128]. Therefore, clinical surrogate markers are needed to reliably predict presence of MVI.

**Figure 2:**
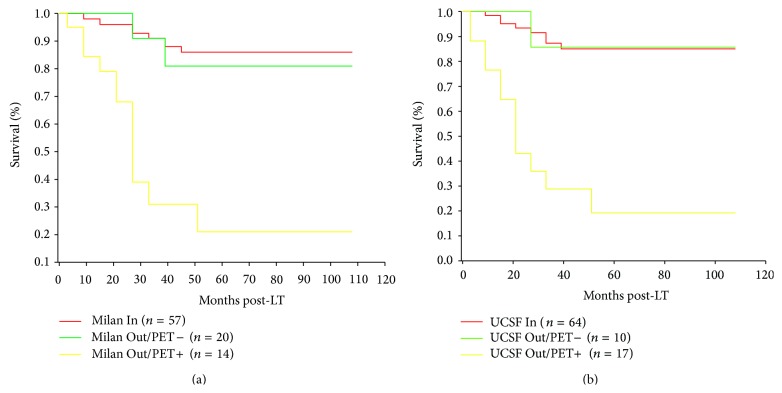
Recurrence-free survival was comparable between Milan In patients and Milan Out (a)/UCSF Out (b) recipients with negative PET scans [135].

Recently, dynamic contrast-enhanced MRI was suggested to be able to indicate MVI [129, 130]. Some other transplant groups have studied the metabolic aggressiveness of HCC by using ^18^F-Fludeoxy (FDG)-positron emission tomography (PET) [131–136]. ^18^F-FDG-PET is nowadays a well-established noninvasive diagnostic tool for the evaluation and treatment monitoring in oncology [137]. The transplant group of Seoul were the first to demonstrate a predictive value of pretransplant PET in the setting of LT for HCC [131] (Table 4). Of 38 liver transplant patients with HCC enrolled, 13 had positive PET scans before LT. The 2-year recurrence-free survival rate of PET− patients was significantly better (85.1%) than in PET+ recipients (46.1%; *P* = 0.0005). Our transplant group was the first to demonstrate a correlation of positive pretransplant PET findings with presence of MVI [132]. In a population of 42 liver transplant patients with HCC, 16 demonstrated increased FDG uptake on pre-LT PET, while 26 patients revealed negative PET findings (Table 4). HCC recurrence rate posttransplantation was 50% in the PET+ group but only 3.8% in PET− recipients. PET+ status was identified as the only independent clinical variable to predict MVI [132]. In a follow-up trial including a larger number of patients, we have recently assessed the prognostic value of pretransplant PET scans in LT for advanced HCC [135]. In a population of 91 patients, we reported about a comparable 5-year recurrence-free survival rate between patients meeting (86.2%) and patients with non-^18^F-FDG-avid HCC exceeding the Milan criteria (81%). In contrast, patients with HCC beyond the Milan size limits and positive PET scans demonstrated a significant worse survival (21%, *P* < 0.001). Remarkably, patients with PET negative HCC beyond the UCSF criteria did also very well, since they had a 5-year recurrence-free survival probability of 85.7% (Figures 2(a) and 2(b)). In multivariate analysis, PET− status of the tumor, AFP-level below 400 IU/mL, and total tumor diameter <10 cm were identified as independent clinical predictors of recurrence-free outcome. We concluded that ^18^F-FDG-PET may be a useful metabolic tool for the evaluation of biological tumor aggressiveness in liver transplant candidates with HCC beyond MC [135]. And, just recently, Lee et al. from the Seoul transplant group reported on the predictive value of ^18^F-FDG-PET in 191 patients after LDLT [136]. Since early tumor recurrence (within 6 months from LDLT) may be associated with less favourable prognosis than late tumor recurrence (beyond 6 months post-LDLT), they focussed on the prognostic impact of ^18^F-FDG-PET to identify patients with those high-risk HCC. A total of 20 and 18 liver recipients developed tumor recurrence early and late after LDLT, respectively. PET+ status was identified as the only independent variable to predict early post-LDLT tumor recurrence [136].

The implementation of biochemical parameters to describe the risk of MVI is another interesting approach [138–140]. In several trials, Des-Gamma-Carboxy Prothrombin (DCP), a protein induced by vitamin k absence or antagonist II (PIVKA-II), has been proposed as important tumor marker in the diagnosis of HCC, especially in Japan. Furthermore, high levels of DCP were shown to indicate vascular invasiveness of the tumor. Shirabe et al. reported on a strong correlation of increased DCP level with risk of MVI in a population of patients with HCC undergoing liver resection or LDLT [138]. Fujiki et al. from Kyoto reported in 2009 on the significance of DCP in selection process of 144 consecutive patients for LDLT [139]. In a multivariate analysis, they identified tumor size >5 cm, number of tumor nodules ≥11, and DCP values >400 mAU/mL as independent predictors of tumor recurrence. For patients with increased DCP levels, incidence of MVI and poor differentiation was significantly higher than for patients with DCP levels ≤400 mAU/mL. Based on these results, the authors created the so-called Kyoto criteria, allowing LT for extended HCC: tumor size ≤5 cm, ≤10 tumor nodules, and DCP values ≤400 mAU/mL. In patients with HCC meeting the MC (*n* = 78) and those exceeding them but meeting the Kyoto criteria (*n* = 28), 5-year recurrence-free survival rates were comparable (7% versus 4%). However, the tumor recurrence rate was significantly higher in patients with HCC beyond the extended Kyoto criteria (*n* = 36; 55%) [139]. The same group had just recently performed a retrospective/prospective validation trial including 198 patients undergoing LDLT for HCC [140]. The 5-year overall survival rates were significantly higher for patients with HCC meeting the Kyoto criteria (*n* = 147; 82%) compared to patients exceeding them (*n* = 49; 42%). The respective 5-year recurrence rates were 4.4% and 51%, respectively. Furthermore, the incidences of MVI and poor tumor grading as parameters of biological tumor aggressiveness were significantly lower in patients within the Kyoto criteria [140].

Alpha-fetoprotein (AFP) is still recognized as the most important tumor marker in patients suffering from HCC [19, 21]. It has not only diagnostic value but also predictive significance. A correlation between increased AFP levels and MVI has already been demonstrated [141–143]. AFP level was shown to provide important information on the selection process of liver transplant candidates with HCC beyond MC [143–153]. Yang et al. from the Seoul transplant group created a new scoring system integrating tumor size, number of tumor nodules, and AFP level based on a series of 63 consecutive patients with HCC undergoing LDLT [150]. According to histopathologic data, the new scoring system correlated well with the risk of HCC recurrence and death, even in patients with HCC beyond MC. Although sample size was rather small, the authors concluded that the new scoring system might effectively expand selection criteria for patients with HCC, without adversely affecting outcome [150]. Ciccarelli and colleagues from Brussels performed a retrospective analysis in 137 liver transplant patients with HCC [151]. In their investigation, AFP level ≥400 ng/mL but not parameters of tumor morphology were identified as pretransplant available independent clinical predictors of HCC recurrence [151]. Other groups pointed out that rather preoperative AFP slope than single AFP value was predictive of posttransplant outcome [144, 147]. Lai et al. on behalf of the European Hepatocellular Cancer Liver Transplant Group reported on 306 patients with HCC meeting and 116 patients with HCC exceeding the MC [144]. The patients underwent locoregional therapies prior to LT. For both, Milan In and Milan Out recipients, AFP slope >15 ng/mL/month, and clinical progression of the tumor under locoregional therapies (as based on modified “response evaluation criteria in solid organs” (RECIST)) were identified as unique independent risk factors for HCC. Based on their results, the authors proposed a very modern criteria system, implementing both radiological and biological tumor behaviours in pretransplant tumor staging [144]. And, just recently, Berry and Ioannou from Seattle calculated the risk of posttransplant death associated with serum AFP level or HCC tumor burden in 45267 adult first liver transplant patients of the USA between 2002 and 2011 [149]. They demonstrated that rather AFP level than macromorphologic tumor burden was the most relevant tumor characteristic to be strongly associated with post-LT outcome. The risk of HCC recurrence was significantly increasing with rising pretransplant AFP values [149].

Apart from established tumor markers, such as AFP and DCP, attention has been focused increasingly on parameters of inflammation. In recent years, the link between poor outcome and systemic inflammation has been demonstrated for several tumor entities [154–157]. Inflammation markers, such as C-reactive protein (CRP), neutrophil-to-lymphocyte ratio (NLR), and platelet-to-lymphocyte ratio (PLR), are increasingly studied in the context of HCC. Elevated parameters of inflammation were recently demonstrated to enhance the risk of recurrence after different treatment modalities of HCC, such as LR, TACE, and RFA, although correlation with MVI has not been consistently shown [157–159]. Halazun et al. reported on the impact of pretransplant elevated NLR (≥5) on tumor recurrence in 150 liver transplant patients with HCC [160]. Elevated NLR was identified in 13 patients. Tumor recurrence post-LT became evident in 62% of patients with increased, but in only 14% of patients without increased NLR (*P* < 0.001), respectively. Milan Out patients with normal NLR had a better recurrence-free outcome than Milan In patients with elevated NLR. NLR and tumor size >3 cm remained the only independent variables to predict tumor recurrence in multivariate analysis [160] (Table 4). Lai et al. have assessed the value of NLR and PLR as predictors of drop-out and post-LT tumor recurrence in a subset of 181 liver transplant candidates [161]. During waiting time, 18 of 181 patients (9.9%) dropped from the waiting list due to HCC-related reasons. The last pretransplant NLR was identified as the best predictor of patients' drop-out, while final PLR better stratified patients in relation to recurrence-free survival. AFP was again identified as excellent parameter to predict both patients' drop-out and HCC recurrence [161]. However, the value of NLR and PLR in the context liver HCC beyond MC has not been analyzed in this trial.

There is accumulating evidence that the prototypical inflammatory cytokine CRP reveals some important prognostic value after different treatment modalities in patients with HCC [162–170]. However, data in the context of LT is still very rare. An and colleagues from the Seoul transplant center have recently evaluated the predictive value of pretransplant CRP level in 85 liver transplant patients with HCC [163]. Increased CRP levels (≥1 mg/dL) were determined in 27 liver transplant patients, while 58 liver recipients had CRP values within normal range (<1 mg/dL) prior to LT. CRP level did not significantly correlate with the presence of vascular tumor invasion. Nonetheless, increased CRP level and MVI were identified as independent predictors of tumor recurrence in patients with HCC exceeding MC. In a subanalysis according to Milan criteria, elevated CRP level and moderate/poor differentiation were the most important predictors of HCC relapse in patients with HCC exceeding but not in patients meeting the MC, respectively. The authors concluded that CRP could be considered as useful and cheap biomarker of tumor aggressiveness and outcome after liver transplantation for HCC, particularly, in patients with HCC beyond MC [163] (Table 4).

Apart from poor tumor grading and MVI as histopathologic parameters to describe tumor aggressiveness, increasing attention is being turned to molecular data for insights into HCC biology. Gene expression studies applying microarray analysis may provide some useful even though preliminary data [171–175]. Their applicability in the transplant setting is, however, still very limited due to high instability of several prognosis-associated genes. Another major issue in this context is that the phenotype HCC may be induced by a considerable heterogeneity of genetic/molecular defects [173, 174]. The prognostic significance of several molecular profiles in the context of LT for HCC is currently under evaluation. Schwartz et al. have analyzed allelic imbalance of 18 microsatellites in 70 consecutive liver transplant patients with HCC (35 patients meeting and 35 patients exceeding MC) [174]. They suggested allelic imbalance in 9/18 microsatellites to correlate with tumor recurrence. Apart from macrovascular invasion, allelic imbalance >0.27 was identified as independent predictor of tumor recurrence in patients with HCC beyond MC (Table 4). Recurrence probability at 5 years after LT was 85% and 10% in Milan Out patients with allelic imbalance > versus ≤ 0.27 (*P* < 0.0002). Jonas et al. from Berlin have recently suggested a selection approach by implementing DNA index [176]. In a series of 246 liver transplant patients with HCC, DNA index was determined at explant histopathology. A DNA index ≤1.5 was detected in 159 patients, while it exceeded 1.5 in 87 patients. There were significant differences in 5- and 10-year survival rates between patients with a DNA index ≤ (86%; 80%) versus >1.5 (27%; 6%), respectively. Five- and 10-year survival rates were excellent in Milan Out patients with a DNA index ≤1.5 (72%; 68%), in contrast to Milan Out recipients exhibiting a DNA index >1.5 (26%, 3%). The authors concluded that the assessment of DNA index is a suitable diagnostic tool to identify patients with advanced HCC that may benefit from LT (Table 4). However, DNA data was based on retrospective explant histopathology reviews and not on pretransplant biopsy. Therefore, the results have to be prospectively validated by implementing pre-LT tumor biopsy results [176].

### 2.2. Neoadjuvant Tumor Treatment

As a result of the increasing numbers of liver transplant candidates and decreasing organ availability, pre-LT waiting times have been persistently prolonged [85, 86]. Effective neoadjuvant tumor therapies are, therefore, mandatory in order to reduce the risk of patients' drop-out and mortality. Apart from LT, techniques TACE and RFA have been considerably improved in recent years and are widely used as “bridging therapies” prior to LT [66, 177, 178]. Nowadays, the practice of treating liver transplant candidates with HCC before or after being set on the waiting list is standard of care in most transplant centers around the world. Traditionally, these neoadjuvant treatments may follow different aims, which are well described in several reviews [77, 179–182] as follows:

- control of tumor growth and prevention of tumor-related drop-out from the waiting list,
- improvement of posttransplant recurrence-free outcome,
- downstaging of advanced HCC into accepted selection criteria.

Apart from that, the capabilities of neoadjuvant treatment to serve as biological selection criteria for liver transplant candidates with HCC beyond standard criteria is increasingly in the focus of critical evaluation.

If being feasible, antiviral treatment in patients with viremic hepatitis B- and hepatitis C-related cirrhosis prior to LT is generally recommended [6–9, 12]. This treatment is at the best aiming at the clearance of viral load, which may be easier achieved in liver transplant candidates with HBV-related than in those suffering from HCV-related cirrhosis. It has been demonstrated that successful perioperative antiviral treatment may not only prevent early post-LT allograft failure but also reduce the risk of posttransplant HCC recurrence [183, 184]. Against this background, clearance of viral loads seems to be a cornerstone in the context of extending selection criteria for HCC. Just recently, Campsen et al. reported on a retrospective analysis of 738 patients with HBV-related cirrhosis that underwent LT at 7 major UC transplant centers. The patients were divided into three eras based on evolving strategies of antiviral therapy (1985–1994, 1995–2004, and 2005–2010). Five-year survival rates in patients with concomitant HBV/HCC were significantly better in era 3 and era 2 with improved antiviral treatment (85.3%, 75.2%) than in patients of era 1 at the beginning of antiviral concepts (31.4%, *P* < 0.001). Similarly, HCC recurrence rates at 5 years were significantly higher in patients of era 3 and era 2 (14.1%, 19.2%) when compared to patients of era 1 (38.1%; *P* = 0.009). Multivariate Cox regression analysis indicated that patients with HBV reinfection were 3.6 times more likely to develop HCC recurrence than patients who did not have HBV reinfection after LT [185]. The authors finally stated that their data justify further attempts at LT for patients with HBV-related cirrhosis and HCC beyond the Milan criteria if the underlying viral disease is aggressively and successfully treated [185]. More data is needed in this field.

Liver resection in patients with HCC on compensated cirrhosis is the longest practiced technique of neoadjuvant “bridging” therapy prior to LT [33, 64, 65, 76, 186]. The decision for conventional surgery is based on tumor size, on topographic location of the nodules, and, above all, on functional liver reserve and the expected waiting time [186, 187]. Besides tumor control, better assessment of relevant histopathologic parameters of tumor biology is the rationale for complete tumor removal [183–188]. In the case of poor prognostic variables assessed at histopathologic analysis (such as MVI and poor tumor grading), early preemptive LT may be advised, before tumor recurrence will occur. In contrast, LT may be postponed, if these histopathologic variables are lacking. Such patients have to be embedded in a concise surveillance program and will be only candidates for LT when liver function is deteriorating or the tumor recurs (the so-called salvage LT) [48, 49, 183–188].

Liver resection as primary treatment for HCC with salvage LT in mind for hepatic deterioration or tumor recurrence has been first described by Majno et al. [189] and proved to be the preferred approach. Several consecutive trials have demonstrated that salvage LT may be effectively performed for patients with HCC recurrence or liver function deterioration after LR [48, 49, 183–193]. Only one initial large study performed by Adam et al. reported about higher operative mortality, an increased risk of posttransplant tumor recurrence, and poorer outcome after secondary versus primary LT [194]. However, there are only few trials that have compared the overall outcome between patients after LR as bridging to LT versus primary LT. Recently, Fuks et al. have demonstrated results of an intent-to-treat analysis in 329 potential candidates for LT with HCC meeting the MC [195]. One hundred and thirty-eight patients with appropriate liver function underwent LR in a perspective of salvage LT for post-LT complications, while 191 were listed for primary LT. Five-year overall survival was not statistically different between the LR-group (77%) and the primary LT-population (60%). However, 51 patients with tumor recurrence after LT (37%) were not eligible for salvage LT. Independent predictors for recurrence-related nontransplantability were MVI, satellite nodules, tumor size >3 cm, poorly differentiated tumor, and liver cirrhosis. The authors concluded that there is a high risk for failure of the concept “salvage LT.” Therefore, this clinical approach should be reserved for those patients with beneficial tumor features assessed after LR [195]. Experiences with salvage LT after LR of HCC primarily exceeding the MC is rare. Facciuto et al. reported on a series of 55 patients with advanced HCC [196]. Twenty-three of them were primarily treated by LR for tumor control; 5 of them eventually underwent salvage LT in early stage recurrence. Primary LT has been performed in 32 patients. Recurrence-free survival was significantly higher after primary LT (65%) than after LR (26%, *P* = 0.01). At a median of 18 months post-salvage LT, all patients were still alive. The authors concluded that, for patients with HCC beyond the MC, a multimodality approach including LR, salvage LT, and primary LT may be useful. However, the number of patients included was rather small and patients' characteristics were very inhomogeneous [196]. A Chinese group has recently reported on their results of salvage LT in 36 patients with recurrent HCC compared to primary LT in 147 patients with HCC meeting and 156 patients with HCC exceeding the MC [197]. Operative complication rate was higher in salvage LT than in primary LT. HCC recurrence rates in the salvage LT-group were significantly lower than after primary LT for Milan Out HCC [197]. And, just recently, another Chinese group reported on their series of 380 liver transplant patients with HCC meeting the UCSF criteria [198]. Two hundred patients underwent LR with a perspective of salvage LT, while 180 patients have received a primary liver allograft. HCC recurrence rate was 43% after LR and 27.2% after primary LT. Only 39 of 86 patients with HCC recurrence after LR were still eligible for salvage LT. Overall survival rate at 5 years was significantly better after primary LT (72%) than in the LR-population (52%; *P* = 0.005). Perioperative mortality and posttransplant complications were comparable between the subgroups. Five-year survival rates were comparable after primary (72%) and salvage LT (61%; *P* = 0.5). The authors concluded that the concept of prior hepatectomy and salvage LT might be useful for patients with HCC beyond MC [198].

Against the background of limited organ availability and advanced tumor stage, the main purpose of studies in this context should be the validation of LR as suitable procedure to select appropriate patients for potential salvage LT. Although data is still limited, there seems to be some good evidence that LR as bridging to salvage LT provides several advantages for patients with advanced HCC and compensated liver function [194–200]. First, it is far less incriminating than primary LT without the need of lifetime immunosuppression and it may be performed without delay. Second, several patients will achieve recurrence-free long-term survival after LR and, thereby, a significant number of allografts may be saved for other patients. And, furthermore, LR is an adequate tool for maintaining transplantability, since advanced HCC carries a high risk of tumor-related drop-out by macrovascular invasion or extrahepatic spread of the tumor [194–200].

**Figure 3:**
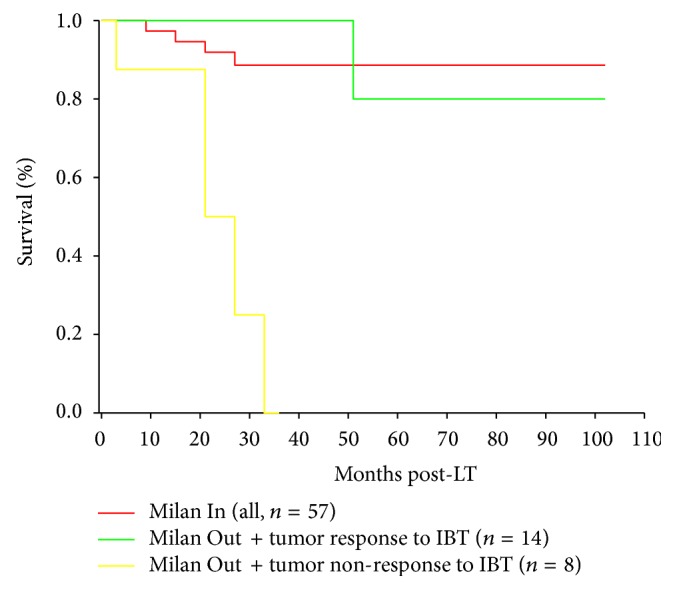
Milan Out patients with adequate postinterventional tumor response on explant histopathology had a comparable survival rate than Milan In patients [213].

In recent years, techniques of TACE and RFA were increasingly implemented as neoadjuvant bridging therapies prior to LT [201, 202]. Nowadays, especially, TACE is locoregional standard of care in those liver transplant candidates, where liver resection is not feasible. Until today, a huge number of studies and reviews report on technique and efficacies of TACE [144, 146, 151, 177, 201–204]. However, there were great differences with respect to study character, number of patients included, intentions of treatments, and criteria for indicating LT and patients' tumor-related drop-out [201–211]. The exact prognostic impact of TACE in the context of LT for HCC is yet still undefined. In an evidence-based analysis, Lesurtel et al. performed an electronic search on Medline database (1990–2005) to identify relevant studies [205]. The selected trials were analyzed and ranked according to the grading system proposed by the Oxford Center for Evidence-based Medicine. Based on this analysis, the authors concluded that there is currently no sufficient evidence that TACE as bridging therapy offers any benefit for liver transplant patients with early or advanced HCC, neither with respect to posttransplant outcome nor to predict pretransplant drop-out [205]. They identified the lack of randomized controlled trials as the main reason for limited validity of previous trials. This may be owed to the fact that most transplant physicians feel obliged to offer their patients any treatment option while waiting for LT [205]. Independent from TACE results, patients with HCC meeting the MC will, per definition, remain suitable candidates for LT, as long as tumor does not exceed conventional criteria limits, invade into major vascular vessels, or spread to the extrahepatic region [64, 65]. In contrast, obvious and reliable TACE-related criteria for achieving transplantability in patients with HCC exceeding MC have not yet been described (Table 2). In a prospective series, Graziadei and colleagues have compared the prognostic impact of TACE in liver transplant patients with early and advanced HCC [206]. None of the patients meeting MC had to be dropped from the waiting list, while drop-out rate was 20% in patients with HCC exceeding them. Posttransplant HCC recurrence rate was 2.4% in the early stage HCC-group but 30% in the advanced HCC-population. The authors concluded that TACE is an effective neoadjuvant treatment approach in patients with HCC meeting but not in those exceeding the MC [206]. In an intent-to-treat analysis, the same team reported on the impact of clinical tumor response to TACE (evaluated on CT scans) on overall survival [207]. A total of 116 patients were included in this trial. The intent-to-treat analysis demonstrated that patients with either complete (no vital tumor on control CT) or partial (devascularisation ≥ 30% on control CT) response to TACE had a significantly better 1-, 2-, and 5-year survival (100%, 93.2%, and 85.7% and 93.8%, 83.6%, and 66.2%, resp.) than patients without adequate response or even tumor progression under TACE (82.4%, 50.7%, and 19.3%). One hundred and six patients have finally undergone LT. Comparably, posttransplant outcome was significantly better in patients with complete or partial response than in those without adequate response to TACE. In a subanalysis according to MC, however, this effect could only be detected in Milan In patients and not in patients exceeding the MC. The authors concluded that patients whose disease fulfilled the MC benefit from TACE if the tumor shows a minimum post-TACE devascularization of 30%, which was not proven for patients with advanced HCC [207]. In contrast to these two studies of the same transplant group, several recent trials were able to demonstrate a beneficial role of TACE in selecting and treating liver transplant patients with HCC beyond MC. Otto and colleagues from Mainz reported on ninety-six consecutive liver transplant patients with HCC that underwent repeated TACE procedures [208]. Sixty-two of them revealed HCC exceeding MC on clinical staging. Finally 50 patients received a liver transplant; 34 of them were beyond MC at pretransplant clinical staging. Five-year survival rate of the entire study group (*n* = 96) was 51.9%. Freedom from recurrence after 5 years was 94.5% in patients (*n* = 39) with progression-free TACE and 35.4% in those with tumor progression despite TACE (*n* = 11; *P* = 0.0017). Recurrence-free survival after 5 years after LT was comparable between patients meeting (93.8%) and those exceeding MC (74.5%; *P* = 0.421). The authors concluded that, by using sustained response to TACE as biological selection criterion, even large and multifocal HCC may be successfully transplanted [208]. Other authors have suggested tumor downstaging by TACE into Milan size limits to produce posttransplant recurrence-free survival rates >50% in patients with HCC initially staged beyond MC [209–211]. In an international consensus conference held in 2010 in London, the current practice of LT in patients with HCC was reevaluated and internationally accepted statements were developed. An overall of 77 statements covering all issues of LT in patients with HCC were established; among them, 5 statements were dealing with the management of waiting list patients. The work stream could not make a recommendation for bridging therapies in patients with UNOS T1 HCC due to lack of evidence. In patients with UNOS T2 HCC (corresponding to “meeting the MC”) likely to wait longer than 6 months for LT, locoregional therapy may be appropriate, although level of evidence is still weak. Albeit no recommendation of using bridging therapies in patients with advanced HCC has been made, there are increasing indices about the value of TACE as biological selection device prior to LT [65]. De Carlis et al. have demonstrated that HCC progression under locoregional bridging therapy was the only independent risk factor for posttransplant HCC recurrence in a population of 118 liver transplant patients with both early and advanced stage HCC [212]. And, just recently, we were able to report on the prognostic significance of postinterventional tumor necrosis in the setting of LT for advanced HCC [213]. In an overall population of 93 liver transplant patients with HCC, we did not assess a survival benefit in 59 of them after neoadjuvant locoregional therapies. However, tumor response following bridging treatments (mainly by TACE) as based on explant histopathology (≥50% tumor necrosis rate) resulted in a significantly better 5-year recurrence-free survival (96%) when compared to patients without adequate tumor response (<50% tumor necrosis rate; *P* < 0.001). Patients with HCC beyond MC on clinical staging but adequate response to neoadjuvant therapies on explant histopathology have achieved an excellent 5-year recurrence-free survival rate of 80%, compared to 0% in those patients exceeding MC and failing to respond (Figure 3). None of macromorphologic parameters but only metabolic tumor uptake pattern on pretransplant PET was identified as independent predictor of postinterventional tumor response. We concluded that postinterventional tumor necrosis promotes recurrence-free outcome in liver transplant patients with HCC exceeding the Milan criteria on clinical staging. Pretransplant PET may be useful in identifying those patients with advanced HCC that will benefit from TACE [213]. Several other trails have recently confirmed the exceptional prognostic value of tumor necrosis/nonviable HCC on explant histopathology for achieving recurrence-free long-term outcome [214].

## 3. Perioperative Approach

Apart from optimized patients' selection by implementation of biological tumor features and application of effective neoadjuvant bridging treatments, the perioperative period offers some further interesting options of beneficial modulation. In the context of the surgical procedure, prevention of tumor cell engraftment into the transplanted liver is the major intention of modulating activities.

### 3.1. “No-Touch” Surgical Technique

The release of tumor cells during liver surgery for malignant tumors is an underestimated problem. A so-called “no-touch” technique has been proposed for successful treatment of big hepatic tumors in the context of LR [215, 216]. The so-called “anterior approach” was demonstrated to provide such a “no-touch” technique of the tumor-bearing liver, which reduces the risk of tumor cell seeding. Although a completely “no-touch” technique in LT may be unrealistic, especially, in those with complicated surgical proceeding, the transplant surgeon must be aware of the risk of setting the nucleus of posttransplant HCC recurrence by inconsiderate surgical acting.

### 3.2. The Role of Living Donor Liver Transplantation (LDLT)

In times of an increasing demand of appropriate liver allografts, LDLT could be in many respects a promising alternative to DDLT [97, 98, 217, 218]. First, live donor liver grafts may theoretically be supplied without limits and be transplanted in an elective approach. Thus, the risk of relevant tumor progression and tumor-related drop-out during a long waiting period might be reduced. Second, LDLT is associated with reduced cold and warm ischemia times and live donor allografts are, thereby, of excellent quality, which could result in better overall outcome. And, third, LDLT with relatives might induce immunological tolerability with less need of immunosuppression and, thus, minimize the risk of posttransplant HCC recurrence [219, 220]. As LDLT is not dependent on regular allocation algorithms, its wide application might take pressure from the pool of deceased donor organs, which could be spared for patients with early-stage HCC or other indications [150, 220]. However, there are some critical issues that have to be addressed in this context.

First, LDLT is a highly sophisticated surgical procedure that puts the donor on a relevant risk of hepatectomy-related morbidity and even mortality [220, 221]. Performing extended LR in a healthy donor implies a relevant ethical dilemma [221]. Therefore, a very concise evaluation of the donor, of donor/recipient matching, and of highly skilled surgical expertise is mandatory [222, 223]. Furthermore, LDLT may from a theoretical point of view be associated with increased risk for posttransplant HCC recurrence and impaired outcome: (1) LDLT represents “fast track” surgery as compared to DDLT, associated with a significant reduction of pretransplant waiting times for LT. The natural selection process and drop-out of patients' with biologically unfavourable HCC could thereby be undermined [217–219]. (2) The partial live donor liver allograft experiences a rapid regeneration process which might promote HCC recurrence in a condition of immunosuppression [222, 223].

In fact, outcome results after LDLT compared to DDLT for HCC are contradictory. Lo et al. from Hong Kong reported on a significantly higher 5-year recurrence rate after LDLT (*n* = 43; 29%) compared to DLDT (*n* = 17; 0%; *P* = 0.029). However, the LDLT-group had fewer incidental tumors and a lower rate of pretransplant TACE but a higher rate of salvage LT, which represents a relevant bias in this trial [224]. Fisher and colleagues have demonstrated a higher HCC recurrence rate but comparable outcome after LDLT versus DDLT [225]. In contrast, other groups did not find a significant difference in outcome data. Bhangui et al. performed an intent-to-treat analysis including 183 consecutive liver transplant candidates, 36 patients for LDLT, and 147 patients for DDLT, respectively [226]. Twenty-seven patients (18.4%) dropped from the waiting list; all of them were scheduled for DDLT. Tumor recurrence rates were comparable between the groups (12.9% versus 12.8%; *P* = 0.78). The overall survival on an intent-to-treat analysis was not different [226]. Two recently performed meta-analyses came to different conclusions. A Chinese group reported on comparable outcome results [227], while colleagues from Toronto demonstrated lower disease-free survival rates after LDLT compared to DDLT [228].

Given that studies are very heterogeneous and mostly of retrospective character, it remains currently unclear if LDLT may provide comparable prognosis than DDLT. However, present data have clearly demonstrated that although biologically unfavourable tumors may be selected by reduced waiting times, LDLT provides acceptable outcome for patients with HCC beyond conventional criteria [228–231] (Table 3). The above-quoted international consensus conference has judged LDLT as ethically acceptable in patients with tumor stages beyond standard criteria, since, in contrast to DDLT, other patients on the waiting list are not negatively affected by this procedure. However, the risk for the donor must be justified by an acceptable prognosis of the recipient. There is still considerable disagreement among the experts involved, what the threshold of posttransplant survival might be. In general, a minimum of 50% survival probability 5 years after LDLT seems to be applicable [65].

### 3.3. The Impact of Ischemia/Reperfusion (I/R) Injury

Ischemia reperfusion (I/R) injury may result in hepatocellular allograft dysfunction, increased rate of ischemic cholangiopathy, and decreased allograft survival with need for liver retransplantation [232, 233]. Although data is still limited, there is some evidence that I/R-associated mechanisms play an important role in the development of intrahepatic tumor recurrence after LT for HCC. In an experimental model, van der Bilt and colleagues from Utrecht have shown that I/R injury is a strong stimulus on the outgrowth of residual intrahepatic colorectal micrometastases, especially, exacerbated in aged and steatotic livers [234, 235]. Man et al. were able to demonstrate that I/R injury of a small liver remnant exacerbated growth of liver tumor and metastases by activation of cell adhesion, invasion, and angiogenesis pathways [236, 237]. The same Chinese group recently succeeded in attenuating I/R injury by application of the immunomodulator FTY720. Its administration resulted in suppression of liver tumor metastasis in an experimental model of LR [236]. Clinical research in this field is still very limited. In a combined clinical and experimental study including 115 liver transplant patients and a rat orthotopic liver transplant model, Ling et al. have shown that posttransplant enhanced signalling of the chemokines CXCL10/CXCR3 in small-for-size grafts induces differentiation and neovessel formation, which further promotes tumor growth. They consequently suggested that targeting these key chemokines may attenuate I/R and, thereby, prevent tumor recurrence after LT [238]. Just recently, Croome et al. presented the results of a clinical investigation using the SRTR to compare the effects of I/R injury in liver allografts donated after cardiac death (DCD, *n* = 242) with livers donated after brain death (DBD, *n* = 5638) [239]. In times of dramatic donor organ shortage, DCD livers are of increasing interest for extending the available organ pool. In contrast to DBD, these allografts suffer from enhanced I/R injury induced by prolonged warm ischemia time because of hypoperfusion and hypoxia during the agonal phase [240]. These organs are consistently exposed to a dual injury by prolonged warm ischemia time and subsequent cold ischemia period. LT by using DCD livers is, therefore, an ideal clinical situation to assess the impact of I/R injury on tumor recurrence. The authors reported on inferior patient and graft survival after transplantation of DCD allografts versus DBD livers. They suggested HCC recurrence to be the main reason for this inferior outcome result since they remained even after adjustment for the inherent inferiority linked to DCD grafts [239].

## 4. Posttransplant Approach

As a result of lifetime need of immunosuppressive therapy, HCC may recur after LT. Early discovery of post-LT tumor relapse may increase the options of curative treatment by surgical procedures [241, 242]. Close surveillance is nowadays standard of post-LT care in liver transplant patients with HCC meeting standard criteria [243]. Since LT for advanced stage HCC might be associated with an increased risk of HCC recurrence, post-LT surveillance program should be intensified in this special subset of patients. However, data about the most optimal post-LT surveillance approach in this context is still lacking. The latest “American Association for the Study of liver Diseases” guidelines on management of HCC did not address the subject of surveillance after curative intent surgery for HCC [4]. The National Comprehensive Cancer Network guidelines for surveillance after curative intent therapy for HCC recommend radiographic imaging every 3–6 months for 2 years, and half yearly thereafter, which should be combined with AFP-level determination [244]. If features of biological tumor aggressiveness have been assessed at histopathology, such as poor tumor differentiation and MVI, an even closer follow-up approach might be justified.

### 4.1. The Role of Immunosuppression

More than 20 years ago, Yokoyama et al. from the Pittsburgh transplant group demonstrated that doubling time of HCC recurrence after LT was significantly shorter than that observed in nonimmunized patients with HCC [242]. The authors concluded that pharmacological immunosuppression in liver transplant patients accelerates growth rates in HCC. Besides, the observation that transplant patients are on higher risk of developing malignancies compared to the nontransplanted population provided some more important indirect evidence of the tumor promoting effects of immunosuppressive therapy [245].

Nowadays, the calcineurin inhibitors (CNI) cyclosporine A (CsA) and tacrolimus (Tac) are the cornerstones of pharmacological immunosuppression in transplant medicine [242, 245]. These immunosuppressive agents affect the T-cell recognition of alloantigen and signal transduction via the calcium-dependent calcineurin pathway. Apart from inhibition of interleukin-2 expression, they promote the expression of transforming growth factor-*β*1, which depresses the natural killer cell-mediated antitumor response and, thereby, promotes the development of metastatic processes [246]. Dantal and colleagues demonstrated in 1998 that increasing CsA dosage leads to an increasing risk for posttransplant de novo malignancies after kidney transplantation [247]. In an experimental model of LT for HCC, Freise and colleagues from UCSF demonstrated CsA to have adverse effects on tumor recurrence [248]. Vivarelli et al. from Bologna were the first to clearly demonstrate a direct correlation of cumulative CsA dosage during first posttransplant year and risk of HCC recurrence [249]. While elevated cumulative CsA dosage had an adverse impact on recurrence-free survival, MC did not impair outcome. The authors concluded that current limits to LT for HCC might be reassessed in view of modified patient management with special regard to immunosuppressive therapy [249]. In several consecutive trials, the same group was able to confirm these data. In 2005, they showed that CsA exposure was the only independent predictor of HCC recurrence. The authors recommended that, in the presence of histopathologic risk factors, individualized immunosuppressive protocols should be considered [250]. Furthermore, they have demonstrated in 2008 that, apart from poor differentiation and MVI, CsA exposure and Tac exposure were independent predictors of HCC recurrence [251]. Just recently, Rodríguez-Perálvarez and colleagues from Spain were focussing on the impact of early post-LT CNI exposure on tumor recurrence. In a population of 219 liver transplant patients with HCC, they were able to demonstrate that reduced exposure to CNI early after LT, where CNI levels are traditionally rather high, may prevent late recurrence of HCC. The authors concluded that reduced dosage immunosuppressive protocols early after LT should be implemented to improve patients' long-term prognosis [252].

While there is sufficient evidence about the benefits of CNI reduction/minimization, the value of steroid-free immunosuppression in this context has not been thoroughly analyzed. A Chinese group recently reported on a series of 178 patients with HCC that underwent LT [253]. All of them have received a Tac-based immunosuppressive regimen containing mycophenolate mofetil and either basiliximab, an interleukin-2-receptor inhibitor (*n* = 78), or steroids (*n* = 100). Overall and disease-free survival rates were comparable between the groups. In a subanalysis of patients meeting MC, however, patients under steroids had a significantly lower overall 5-year survival (57.4%) compared to those receiving basiliximab (88.9%; *P* = 0.022). These interesting results have to be further validated.

In recent years, there is increasing interest in a new category of immunosuppressive drug, the so-called m-TOR (mammalian target of rapamycin) inhibitors [254, 255]. The immunosuppressive efficacy of this drug is a result of its capability to block interleukin-2 stimulation of lymphocyte proliferation. Furthermore, it is supposed to have anticancer efficacies due to impairment of vascular endothelial growth factor production [256, 257]. There are several reports demonstrating efficacy of m-TOR inhibitors to reduce the incidence of de novo malignancies or even regression of posttransplant neoplasms. Initially, the m-TOR inhibitors sirolimus and everolimus were tested regarding their applicability for CNI-sparing immunosuppression [258]. In 2004, however, Kneteman et al. suggested that a sirolimus-based immunosuppressive regimen may provide beneficial effects on recurrence and outcome in patients with HCC beyond MC [114]. A total of 40 patients with HCC were included in this trial, nineteen of them meeting and 21 recipients exceeding MC, respectively. All of them have received a sirolimus-based immunosuppressive regimen, designed to minimize CNI exposure. There were no significant differences in one- and 4-year survival rates between the Milan In group (94.1% and 87.4%) and the Milan Out population (90.5% and 82.9%). Five patients developed tumor recurrence, one in the Milan In group and 4 patients of the Milan Out population [114]. Subsequently, many transplant centers around the world have implemented a sirolimus-based immunosuppressive regimen in treatment of liver transplant patients with HCC. Unfortunately, this has mostly been done in a noncontrolled monocentric fashion. There are only few reports that have compared a sirolimus-based concept with conventional immunosuppressive regimens [259]. Nevertheless, evidence about the beneficial value of sirolimus-based immunosuppression in liver transplant patients with HCC is continuously increasing in recent years. In 2010, Vivarelli et al. reported about the results of a matched-cohort study including 31 patients under a sirolimus-based regimen and 31 patients receiving Tac-based standard immunosuppression [259]. They reported about significantly better 3-year survival rate in the sirolimus-group (86%) compared to the Tac-population (56%; *P* = 0.04), although risk profiles with respect to MVI, poor tumor grading, and AFP levels were not different between the patients [259]. Toso et al. from Edmonton reported on their study based on the SRTR, including 2491 liver transplant patients with HCC and 12167 with non-HCC disease [260]. In multivariate analysis, only anti-CD25 antibody induction therapy and sirolimus-based immunosuppression were identified as independent predictors of beneficial survival after LT [260]. Furthermore, 2 meta-analyses have recently demonstrated lower tumor recurrence rates and improved survival under a sirolimus-based immunosuppressive regimen when compared to sirolimus-free treatments [261, 262]. On the basis of these hopeful reports, the transplant community is currently waiting for results of the first prospective randomized open-label trial comparing sirolimus-containing versus m-TOR-inhibitor-free immunosuppression in liver transplant patients with HCC (SILVER study) [262].

### 4.2. The Role of Adjuvant Therapy

Adjuvant chemotherapy failed to demonstrate a survival benefit after LR for HCC, since sensitivity of HCC cells to cytostatic drugs is rather low. The first relevant report of adjuvant chemotherapy in the setting of LT was presented by Olthoff et al. in 1995 [263]. A series of 25 patients received intravenous fluorouracil, doxorubicin, and cisplatin for 6 months after LT. Many of them had advanced tumor stages with tumor size ranging from 2 cm up to 20 cm. Six patients did not complete the therapy due to severe side effects. Overall 3-year survival rate was 46% and, thus, significantly better than in a historic control group of 17 patients (5.8%; *P* = 0.0001). The authors concluded that adjuvant chemotherapy after LT for HCC might improve outcome, even in advanced-stage HCC [263]. In 2002, Roayaie and colleagues from New York published data of an uncontrolled prospective study on 43 liver transplant patients with advanced HCC [264]. At the time of LT, they had tumors >5 cm of diameter and have received 6 cycles of systemic doxorubicin after transplantation. In 11 liver recipients, adjuvant chemotherapy had to be finished, mainly for adverse effects. Five-year recurrence-free survival rate was 44% [264].

In contrast to these two uncontrolled studies, more recent trails were not able to demonstrate a beneficial impact of adjuvant treatments [265, 266].

Recently, some hopes on a new drug have been built. Sorafenib is a multi-tyrosine kinase inhibitor and angiogenesis inhibitor that has been approved for the treatment of advanced HCC in 2007 [267]. In experimental models of LR and LT, sorafenib was demonstrated to suppress and delay recurrence of HCC [66, 266]. There are only few clinical studies investigating efficacy of sorafenib on HCC recurrence after LT. In most of them, relevant drug-related adverse effects leading to dose reduction have been reported [66, 266]. Saab et al. from UCLA reported 2012 results on a retrospective case control matched study [268]. Eight liver transplant patients with advanced HCC (based on MC, poor tumor differentiation, and lymphovascular invasion) who tolerated adjuvant sorafenib therapy were matched with patients who did not receive sorafenib. After a mean follow-up of 17.75 ± 6.26 months, 1 of 8 patients treated with sorafenib (12.5%) developed HCC recurrence. In contrast, 4 of 8 matched controls (50%) were suffering from tumor recurrence after a mean follow-up of 31.63 ± 22.30 months. The authors concluded that sorafenib therapy might be safe and result in reduction of HCC recurrence rate after LT [268]. Recently, a Chinese group demonstrated a case control study including 17 liver transplant patients with HCC beyond MC [269]. Eleven of them have received sorafenib as adjuvant therapy after LT. The recurrence-free survival rates for patients with and without sorafenib at 18 months were 66.7% versus 0% (*P* = 0.011). Nine patients were requiring dose reduction due to adverse effects. The authors suggested that sorafenib might improve outcome in liver transplant patients with HCC beyond MC [269]. Prospective randomized trails are needed for further appraisal.

Another Chinese group have reported their experience with a clinical-experimental approach of oncolytic adenoviral therapy in a population of 45 liver transplant patients with HCC beyond Milan criteria [270]. Twenty-two patients underwent LT only, while 23 liver recipients additionally underwent adenovirus-mediated herpes simplex virus thymidine kinase therapy (ADV-TK), which is a well-studied approach for tumor cell eradication. The recurrence-free survival rates at 3 years were significantly better in the LT + ADV-TK group (69.6%) compared to the LT alone population (43.5%; *P* = 0.001). Only vascular invasion was identified as independent predictor of poor outcome. All patients with vascular tumor invasion developed tumor recurrence, which was, however, delayed in the treatment group. In the nonvascular invasion subpopulation, 10 of 12 patients in the LT only and 2 of 12 patients in the LT + ADV-TK subset relapsed. The authors concluded that HCC patients without vascular invasion could be eligible for LT, regardless of tumor size and multifocality, when followed by an ADV-TK treatment [270].

A recent, very detailed review about adjuvant approaches after LT for HCC came finally to the conclusion that, based on data thus far, adjuvant treatment may currently not be recommended, except in the context of trials [266].

## 5. Conclusions

**Figure 4:**
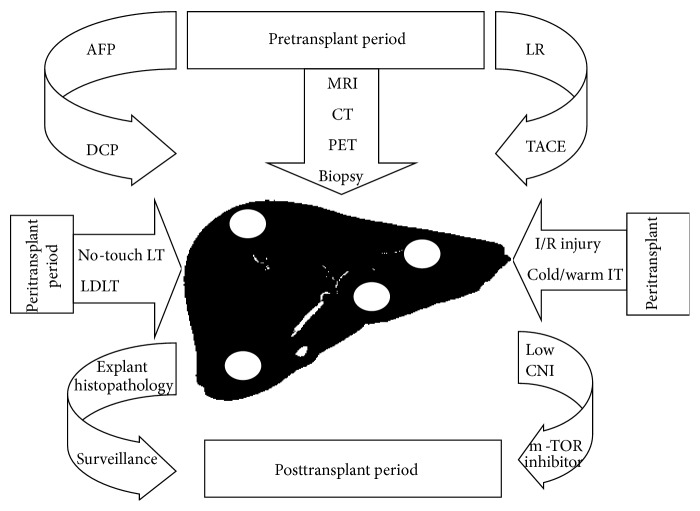
In order to achieve acceptable prognosis, a close multidisciplinary clinical approach in the pre-, peri-, and posttransplant period has to be established.

Based on current data, general exclusion of patients with HCC beyond MC from LT is no longer justified. In fact, the implementation of the MC in 1996 had a tremendous impact on establishing HCC as major indication for LT. Patients with HCC meeting the MC may achieve 5-year recurrence-free survival rates of about 70%. In the last two decades, no other development in the field of visceral surgical oncology was able to provide comparable rates of cure. In recent years, however, huge advancements in radiographic and interventional techniques, surgical procedures, immunosuppressive treatments, and, not least, the understanding of tumor biology have been made. These proceedings opened up new perspectives “beyond” MC, where patients with advanced HCC may be identified to benefit from LT. Posttransplant 5-year survival probabilities above 50% in these patients have intensified the call for an extension of transplant selection criteria. It is a major problem in our days that frequency of organ donation is continuously decreasing and waiting times prior to LT are steadily increasing. Liver transplant candidates with HCC are, thereby, exposed to a significant risk of tumor-related drop-out from the waiting list or posttransplant HCC relapse. Against this background, implementing LT in patients with HCC beyond Milan criteria into clinical routine depicts a multisciplinary challenge (Figure 4).

It is undoubted that adequate patients' selection should be rather based on tumor biology than on static macromorphology of HCC. Histopathologic parameters of aggressive tumor behaviour, such as poor tumor grading or MVI, may be safely and reliably assessed only at explant analysis. Therefore, reliable clinical surrogate markers, such as AFP and DCP, should be incorporated into pretransplant decision process. Since biological tumor aggressiveness may change during prolonged waiting time, tumor biology should be reassessed in a dynamic way. This may be easily performed with already established tumor markers, such as AFP and DCP. Applicability and prognostic importance of genetic profiling and inflammatory parameters in the selection process prior to LT are still a matter of clinical investigation. In contrast, there is increasing evidence that PET assessment of metabolic tumor behaviour is feasible and provides useful prognostic information, especially, in patients with advanced HCC stages. Not least regarding our own experiences, PET evaluation should be part of the dynamic selection process. Neoadjuvant locoregional treatment is currently standard of care in the pretransplant period. In particular, in patients with HCC beyond MC, TACE should not only be used as bridging treatment for tumor control but also be used as biological selection criterion for final decision making. There seems to be enough evidence that tumor progression under TACE identifies those aggressive HCCs that put the patients' on very high risk of post-LT tumor relapse. In contrast, clinical and histopathologic response to pretransplant locoregional intervention was shown to be a useful indicator of beneficial tumor biology, finally justifying LT in HCC stages beyond MC.

Well-considered and anticipatory surgical procedures using tumor “no-touch” techniques and minimizing I/R injury are practical approaches for a beneficial peritransplant modulation. Although clinical data is still rare, cold and warm ischemia times should be kept as low as possible in this special subset of patients. If perioperative pharmacological immunomodulation might reduce the allografts' susceptibility for HCC relapse is still under experimental and clinical investigation.

A close surveillance program should be the cornerstone of the posttransplant period, especially, in those patients where parameters of aggressive tumor biology have been assessed at explant histopathology. The options of beneficial immunomodulation after LT have been underestimated in recent years. Based on current data, however, reduction of immunosuppressive drug exposure is regarded as a major issue of posttransplant period. Furthermore, immunosuppressants with antitumor capabilities, such as m-TOR-inhibitors, have to be implemented, although final confirmation by the SILVER study is still pending. Currently, there is no established effective adjuvant treatment after LT.

In summary, the combination of a dynamic biology-related pretransplant selection process with multidisciplinary pre-, peri-, and posttransplant approaches of tumor and patient modulation contributes to acceptable prognosis of liver transplant patients with HCC beyond standard criteria.

## Figures and Tables

**Table 1:** Reported 5-year survival rates in patients undergoing liver transplantation for HCC meeting proposed extended criteria (based on pretransplant versus posttransplant staging).

Author (year)	Proposed criteria	Pre-LT staging	Explant histology
Yao et al. (2001) [88]	One tumor ≤6.5 cm or ≤3 tumors each ≤4.5 cm Total tumor diameter ≤8.5 cm		75.2%

Cillo et al. (2004) [94]	Any size and number No poorly differentiated tumor	75%	

Kneteman et al. (2004) [114]	One single tumor <7.5 cm or any number <5 cm Sirolimus as immunosuppressant	82.9% (4-year-survival)	

Ito et al. (2007) [95]	≤10 tumor nodules Tumor diameter ≤5 cm PIVKA ≤400 mAU/mL (LDLT)	86.7%	

Onaca et al. (2007) [115]	One tumor ≤6 cm or ≤4 tumors each ≤5 cm	>60%	

Lee et al. (2008) [96]	≤6 tumor nodules Tumor diameter ≤5 cm No gross vascular invasion (LDLT)		76.3%

Herrero et al. (2008) [97]	One tumor ≤6 cm or ≤3 tumors ≤5 cm No macrovascular invasion No extrahepatic spread	70%	

Zheng et al. (2008) [113]	Total tumor diameter ≤8 cm or total tumor diameter >8 cm, Well differentiated, and AFP ≤400 ng/mL No macrovascular invasion		72.3%

Mazzaferro et al. (2009) [98]	Sum of size (largest tumor) and number of nodules = 7 (up-to-seven) No microvascular invasion		71.2%

Muscari et al. (2009) [99]	≤5 tumor nodules Tumor diameter ≤5 cm	77%	76%

Fujiki et al. (2009) [139]	≤10 tumor nodules Tumor diameter ≤5 cm DCP values ≤ 400 mAU/mL	89%	

Dubay et al. (2011) [100]	Any size and number No poor differentiated tumor	72%	

**Table 2:** Results of TACE as neoadjuvant therapy prior to LT in patients with HCC initially beyond MC.

Author (year)	Inclusion criteria	Transplant criteria	Outcome after LT
Graziadei et al. (2003) [206]	Beyond Milan (*n* = 15)	≥50 tumor regression under TACE	31% (intent-to-treat) 5-year overall survival

Millonig et al. (2007) [207]	Within UCSF Beyond UCSF	No progression beyond UCSF ≥50 tumor destruction	66.6% (response)/25% (progression) 25% 5-year overall survival

Otto et al. (2006) [208]	Beyond Milan (*n* = 34)	Tumor regression under TACE	74.5% 5-year overall survival

Ravaioli et al. (2008) [209]	Single HCC nodule ≤8 cm or bifocal HCC ≤5 cm or <6, each ≤4 cm, TTD ≤ 12 cm No macrovascular invasion	Downstaging into Milan criteria (TACE or resection or RFA)	75% 3-year recurrence-free survival

de Luna et al. (2009) [210]	Beyond Milan criteria	Downstaging into Milan criteria	78.8% 3-year overall survival

Jang et al. (2010) [211]	Beyond Milan criteria	Downstaging into Milan criteria	66.3% 5-year recurrence-free survival

**Table 3:** Results of LDLT in patients with extended criteria HCC.

Author (year)	Transplant criteria	Outcome after LT
Bhangui et al. (2011) [226]	Beyond Milan No macrovascular invasion No extrahepatic spread	52.6% 3-year recurrence-free survival

Lee et al. (2008) [96]	≤6 tumor nodules Maximum tumor diameter ≤5 cm No gross vascular invasion	76.3% 5-year overall survival

Florman and Miller (2006) [223]	Any tumor number Maximum tumor diameter <5 cm DCP <300 mAU/mL	80% 5-year recurrence-free survival

Lo et al. (2007) [224]	≤6 tumor nodules Maximum tumor diameter ≤5 cm No gross vascular invasion	76% 5-year recurrence-free survival

Fisher et al. (2007) [225]	≤7 tumor nodules Maximum tumor diameter ≤7 cm	73.4% 5-year survival rate

**Table 4:** Novel approaches of extending selection criteria.

Author (year)	Selection criteria	Outcome after LT
Yang et al. (2006) [131]	^ 18^F-FDG-PET (negative versus positive)	12% recurrence rate PET− patients 36.5% recurrence rate PET+ patients

Kornberg et al. (2009) [132]	^ 18^F-FDG-PET (negative versus positive)	11.1% recurrence rate Milan Out/PET− 53.8% recurrence rate Milan Out/PET+

Kornberg et al. (2012) [135]	^ 18^F-FDG-PET (negative versus positive)	5-year recurrence-free survival: 86.2% Milan In 81% Milan Out/PET− 21% Milan Out/PET+

Halazun et al. (2009) [160]	NLR (< versus ≥5)	5-year recurrence-free survival: 75% in NLR <5 25% in NLR ≥5

An et al. (2012) [163]	CRP (< versus ≥1 mg/dL)	CRP independent predictor of outcome HR 4.64 recurrence-free survival HR 2.68 overall survival

Schwartz et al. (2008) [174]	Allelic imbalance (≤ versus >0.27)	Tumor recurrence probability at 5 years: 10% in AI ≤0.27 85% in AI >0.27

Jonas et al. (2009) [176]	DNA index (≤ versus >1.5)	5- and 10-year survival rate in Milan Out 72% and 68% in DNA ≤1.5 26% and 3% in DNA >1.5

## Abbreviations

HCC: Hepatocellular carcinoma
HBV: Hepatitis B virus
HCV: Hepatitis C virus
MC: Milan criteria
AFP: alpha-fetoprotein
CT: Computed tomography
MRI: Magnetic resonance tomography
MVI: Microvascular invasion
LT: Liver transplantation
LR: Liver resection
BCLC: Barcelona Clinic Liver Cancer
RFA: radiofrequency ablation
TACE: Transarterial chemotherapy
UNOS: United Network for Organ Sharing
MELD: Model for end-stage liver disease
DLDT: Deceased donor liver transplantation
UCSF: University of California San Francisco
UCLA: University of California Los Angeles
^18^F-FDG-PET: ^18^F-Fludeoxy-positron emission tomography
CRP: C-reactive protein
NLR: Neutrophil-to-lymphocyte ratio
PLR: Platelet-to-lymphocyte ratio
I/R: Ischemia reperfusion
SRTR: Scientific Registry of Transplant Recipients
DCD: Donated after cardiac death
DBD: Donated after brain death
CNI: Calcineurin inhibitor
CsA: Cyclosporine A
Tac: Tacrolimus
RECIST: Response evaluation criteria solid organs
FAI: Fractional allelic imbalance
m-TOR: Mammalian target of rapamycin.
